# How far in the future can we predict others’ affective states?

**DOI:** 10.1192/j.eurpsy.2021.370

**Published:** 2021-08-13

**Authors:** E. Cappello, G. Lettieri, G. Handjaras, E. Ricciardi, P. Pietrini, L. Cecchetti

**Affiliations:** 1 Momilab - Sane Group, IMT School for Advances Studies Lucca, Lucca, Italy; 2 Momilab - Sane Group, IMT School for Advanced Studies Lucca, Lucca, Italy; 3 Momilab, IMT School for Advanced Studies, Lucca, Italy

**Keywords:** emotion, Emotion Dynamics, social cognition, theory of mind

## Abstract

**Introduction:**

Human social interactions are rooted in the ability to understand and predict one’s own and others emotions. Individuals develop accurate mental models of emotional transitions (MMET) by observing regularities in affective experiences (DOI: 10.1073/pnas.1616056114) and a failure in this regard can produce maladaptive behaviors, one of the hallmark features in several psychiatric conditions.

**Objectives:**

To investigate whether MMET are stable over time and which emotion dimensions (e.g., valence, dominance) influence MMET over time.

**Methods:**

We selected thirty-seven emotion categories (DOI: 10.1177/0539018405058216) and five different time intervals (from 15 minutes to 4 days). Sixty-two healthy participants rated the likelihood of transition between all possible pairs of affective states at each time interval.

**Results:**

As expected, we observed a trend toward uncertainty as the timescale increased. In addition, the probability of shifting between two affective states having the same valence (e.g., happiness and contentment) was rated higher than for emotions with opposite polarity (e.g., happiness and sadness). Even though this pattern becomes gradually noisier for predictions far in the future, it is still present for infradian intervals (Fig.1).

**Figure:**
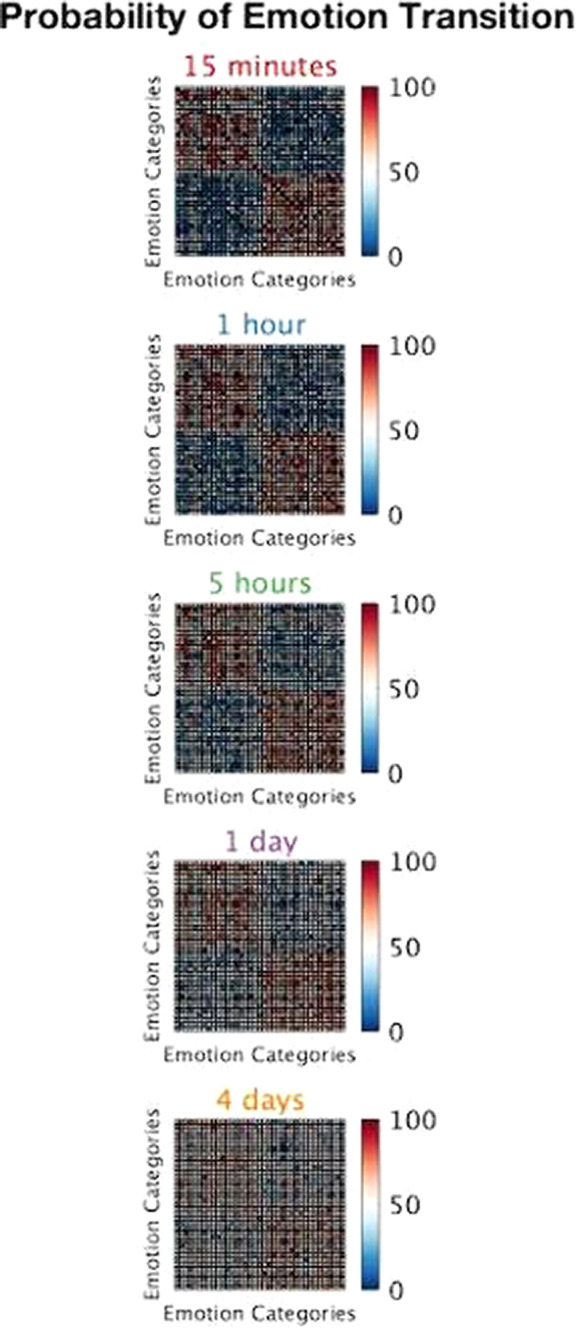

**Conclusions:**

Our results suggest that MMET are informed by the valence dimension and moderately influenced by the timescale of the prediction. These findings in the healthy population may prompt the exploration of emotion dynamics in psychiatric conditions. Future studies could leverage the MMET approach to test whether specific psychiatric disorders (e.g., bipolar disorder) are associated with abnormal patterns of emotion transitions.

**Disclosure:**

No significant relationships.

